# Morning clock gene expression in young adults of early and late chronotypes

**DOI:** 10.1038/s41598-025-12423-7

**Published:** 2025-07-23

**Authors:** Bettina Krueger, Luisa Sophie Rajcsanyi, Katharina Hundertmark, Bianca Stutz, Anke Hinney, Anette Buyken

**Affiliations:** 1 Institute of Nutrition, Consumption and Health, Faculty of Natural Sciences, Paderborn University, Paderborn, Germany; 2 Section of Molecular Genetics in Mental Disorders, LVR-University Hospital Essen, University of Duisburg-Essen, Essen, Germany; 3 Institute of Sex and Gender-Sensitive Medicine, Medical Faculty, University of Duisburg-Essen, Essen, Germany; 4 Center for Translational Neuro- and Behavioral Sciences, University Hospital Essen, Essen, Germany

**Keywords:** Clock genes, Chronotype, Expression level, Young adults, Genetics, Molecular biology, Biomarkers, Medical research

## Abstract

Variations in circadian rhythm-related genes influence the individual chronotype. Here, we hypothesize that the peak of clock gene expression at 7 a.m. differs between young adults with a late chronotype and young adults with an early chronotype. Participants of the Chronotype and Nutrition nutritional trial (ChroNu study) were selected for their chronotype assessed by the Munich Chronotype questionnaire (MCTQ) and actigraphy. Total RNA was isolated from CD14^+^ monocytes of participants at 7 a.m. on the run-in day. Expression levels of seven clock genes (*PER1*,* PER2*,* PER3*, *NR1D1*,* NR1D2*, *CRY1* and *CRISPLD2*) of individuals with early (*n* = 11) or late chronotypes (*n* = 19) were analysed by reverse transcription quantitative polymerase chain reaction. Difference in expression levels was tested by Mann Whitney-U test. The relative expression levels of the selected genes were not significantly different between individuals with early and late chronotypes (all *p* > 0.07). Contrary to expectation, clock gene expression levels at 7 a.m. was similar in individuals with early and late chronotypes. Further studies on larger sample sizes with multiple sampling time points should elucidate whether gene expression is altered at other day times underscoring the biological difference between individuals with early or late chronotypes.

**Supplementary Information:**

The online version contains supplementary material available at 10.1038/s41598-025-12423-7.

## Introduction

Sleep-wake timing, overall activity and many physiological processes are affected by the individual chronotype. The individual chronotype is determined by age, sex as well as genetics. The central circadian „clock“ is located in the suprachiasmatic nucleus (SCN) which receives information on the day-night cycle from the eyes. On a gene expression level, this circadian clock is mainly driven by the circadian expression of the transcription factors *Clock* and *BmalI*, which activate further clock genes *Per1*,* Per2*,* Per3*, *Cry1 and Cry2*, which vice versa inhibit the transcription of *Clock* and *BmalI*. This feedback loop regulates additional genes and many physiological processes in the body^1–4^, e.g. glucose metabolism and insulin sensitivity^5^. From animal models, it is known that molecular clocks exist in cells of peripheral organs e.g. liver, olfactory bulbs, skin, blood cells and pancreas^6^. To synchronize hormone secretion and metabolic processes, peripheral organs adapt to signals from the SCN and the environment^7,8^ in an organ-specific manner. For example, peripheral clocks in the gut are involved in glucose uptake, while they regulate insulin sensitivity in muscle, liver and adipose tissue^5^. Therefore, the circadian rhythm of metabolic processes might depend mainly on the current mRNA expression level of clock genes. Therefore, a chronotype-specific mRNA transcription is likely.

So far, little is known about chronotype-specific clock gene expression levels. Genome-wide association studies (GWAS) have already identified several genetic loci associated with certain chronotypes. The most recent identified 351 genetic loci linked to the chronotype^9–12^ with 15 genes being associated with morning types^10,12^. Hence, clock gene expression patterns might give an indication of the chronotype. Wittenbrink et al. (2018) demonstrated that under routine protocol conditions, with constant light and environmental factors for all participants, twelve genes oscillate in a circadian manner. These genes include main regulators of circadian regulation, such as *PER1*, *PER2*, *PER3*, *CRY1*, *NR1D1*, *NR1D2*^13^. Based on these data, they developed an algorithm to estimate the individual internal circadian time and chronotype. The idea to develop a test kit for the prediction of an individual’s chronotype based on the analyses of only the fundamental circadian transcription factors *BMALI* and *PER2*^14,15^ or *NR1D1*, *NR1D2* and *PER2*^16^ has been further developed by other groups. However, the circadian oscillation of clock genes seems to be crucial when determining the actual expression level of certain clock genes to predict the individual chronotype^13–15,17^. The regular collection of samples at fixed intervals compensates for the oscillatory expression.

To our knowledge, there are only little data so far, investigating chronotype-specificity of clock gene expression from blood cells at one fixed timepoint. Recent data suggest that analysing RNA from hair follicle cells at a single time point may be sufficient to obtain a gene expression pattern^18^. If gene expression is affected by acute misalignment, e.g. a very early blood withdrawal from persons with a late chronotype, this should be seen when comparing expression levels between persons with questionnaire-determined early and late chronotypes by different clock gene expression levels. Indeed, recent data suggest that the relative expression level might be phase shifted in late chronotypes in comparison to early chronotypes because they have a delayed circadian rhythm^16,19^. A correlation of the peak expression of *PER1*, *PER2* and *NR1D1* in persons with early and late chronotypes with midsleep point and melatonin secretion support the idea of phase-shifted peak expression^19^. A chronotype-specific expression pattern of several clock genes might be suitable feature to characterize chronotypes more precisely. In this pattern the maximal mRNA transcription in persons with late chronotypes could be earlier or later than in persons with early chronotype (Fig. S3).

To date, chronotype is commonly assessed by two well established questionnaires: The Munich Chronotype Questionnaire (MCTQ) asks for sleep onset and wake up times to calculate the midpoint of sleep^20^, while the MEQ (morningness-eveningness questionnaire) asks for preferences for morning or evening times^21^. Both questionnaires, therefore, distinguish persons being mainly active in the first part of the day (early chronotype/morning type) from those being active in the second part of the day (late chronotypes/evening types^20,21^.

The ChroNu study, in which persons with the earliest and latest chronotypes were selected from a baseline cohort to participate in a cross-over nutritional trial^22^ offers such a possibility. In this study, blood samples were taken at around 7 a.m., a time at which persons with late chronotypes may experience an acute circadian misalignment after a weekend in which they were allowed to live according to their inner clock, whereas persons with early chronotypes will experience no or a minor circadian misalignment at this timepoint. The hypothesis of this secondary analysis of the ChroNu study was that persons with early and late chronotypes differ in the relative expression levels of clock genes (*PER1*,* PER2*,* PER3*, *CRY1*, *NR1D1*,* NR1D2* and *CRISPLD2*) at the single time point of 7 a.m.

## Methods

### The ChroNu study

**Fig. 1:**
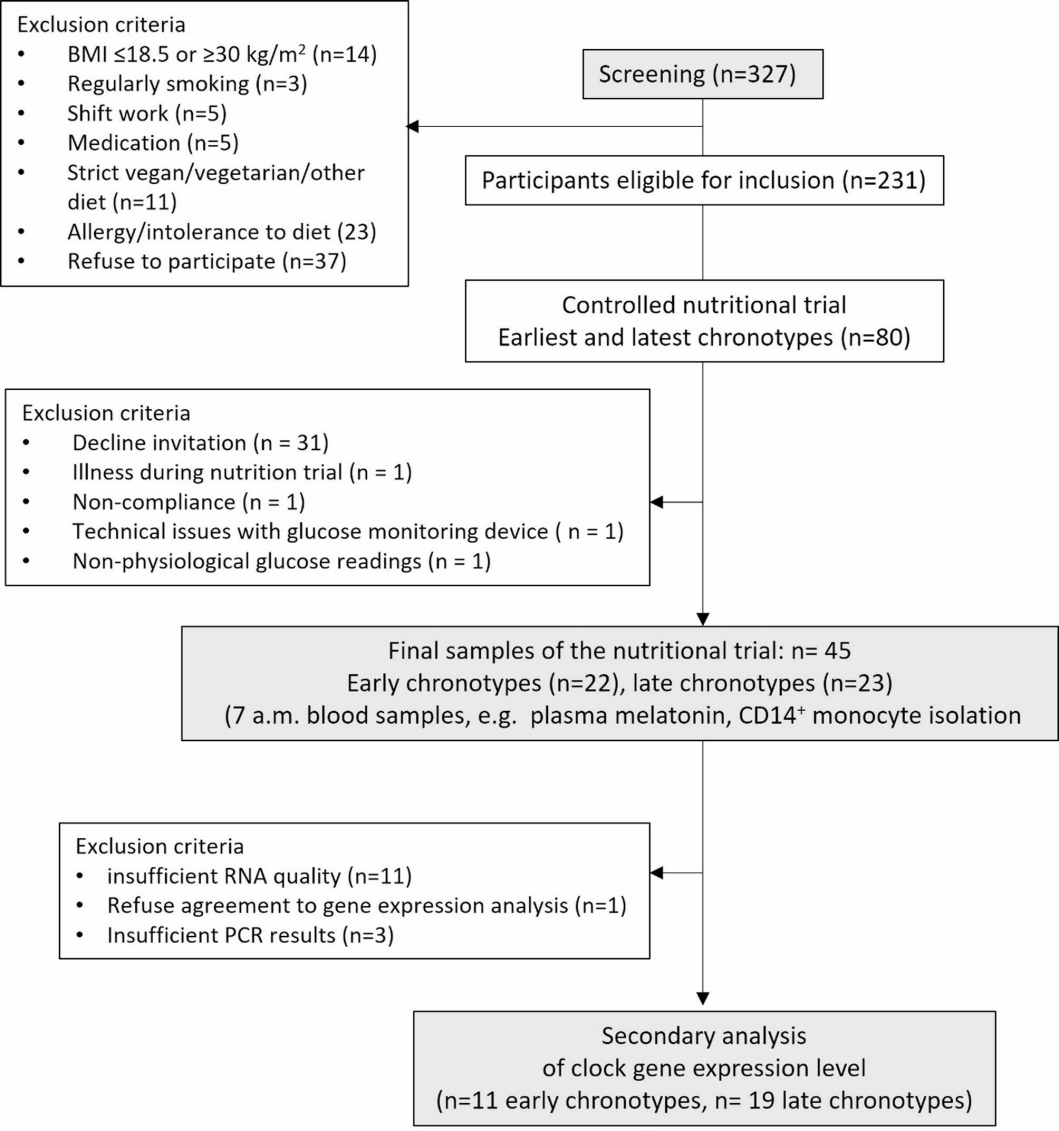
Flow chart of ChroNu study participants and the procedure of the controlled nutrition trial.

The present study is a secondary analysis of a controlled nutrition trial, the Chronotype and Nutrition (ChroNu) study (Fig. 1). Participants were students from all faculties of Paderborn University aged 18–25 years enrolled in a chronotype screening. Exclusion criteria for participation in the subsequent nutrition trial were BMI ≤ 18.5 or ≥ 30 kg/m^2^, regular smoking, strict diet, allergy/intolerance to the intervention diet (see below), pregnancy or lactation, shift work in the past 3 months, crossing of > 1 time zone in the previous 3 months, intake of sleep affecting medications such as antidepressants and sedatives. Students filled out questionnaires on daily routines, physical activity and chronotype^23,24^.

From 231 included persons, those with the earliest (*n* = 40) and the latest (*n* = 40) chronotypes were invited to participate in a randomized controlled nutrition trial with a cross-over design conducted from September to December 2020. Of these, 45 subjects with early (*n* = 22) and late chronotypes (*n* = 23) were included into the RCT. Informed consent was obtained from all participants prior to the trial. The study was performed in accordance with the Declaration of Helsinki^25^ and the study protocol was approved by the Ethics Committee of Paderborn University. The main trial was registered at clinicaltrials.gov (NCT04298645).

Figure 1 illustrates the reasons for non-participation (decline of the invitation, drop-out due to illness, non-compliance, technical issues with the glucose monitoring device and unphysiological glucose readings). For the present secondary analysis of gene expression differences between individuals with early and late chronotypes, EDTA-whole blood samples were analysed from the remaining included participants. 11 RNA extractions did not yield sufficient material for further processes and three RNA samples did not meet quality criteria for qPCR experiments. RNA samples were available from 32 persons (Fig. 1).

Of *n* = 327 screened students, *n* = 80 were invited to participate of which *n* = 46 completed the intervention. *N* = 33 met the criteria for inclusion into the gene expression secondary analysis (for PCR quality criteria see method section). Abbreviations: BMI – body mass index, GI – glycemic index, n – number of participants.

### Chronotype assessment

Chronotype was assessed by the Munich Chrono-Type Questionnaire (MCTQ). The MCTQ inquires bedtimes and wake up times on work and free days within the last four weeks^20^. Chronotype is defined as the midpoint of sleep on free days corrected for sleep debt on work days (MSFsc)^26^. Accelerometery data were used to verify the chronotype using estimated sleeping time and wake up times on the weekend before the intervention. Additionally, participants documented times for sleep onset and wake up in a diary.

### Anthropometric data

Body composition was measured before and after nutrition trial by BIA (SECA mBCA 515), a validated method^27^ estimating body fat mass (%), visceral fat mass (L), and skeletal mass (kg). In addition, body weight (kg) and height (m) were measured using the ultrasonic measuring station (seca 287 dp) from SECA, allowing for the calculation of body mass index (BMI).

### Laboratory measurements

On the run-in day, EDTA whole blood samples were collected at 7 a.m. after ≥ 10 h overnight fast. Plasma and serum extracted from the blood samples were sent for fasting plasma insulin and blood glucose measurements to the German Diabetes Center, Düsseldorf, Germany. Fasting plasma insulin was measured by chemiluminescence immunoassay (Immulite 2000 xPi; Siemens), while fasting blood glucose (hexokinase reference method) was measured by an enzymatic colorimetric assay (Cobas c-311; Roche). Serum samples were re-analysed for morning melatonin levels from samples for the secondary analyses by ELISA assays (Tecan IBL International GmbH, Hamburg) with the sunrise ELISA reader (Tecan IBL International GmbH, Hamburg) at the German Diabetes Center, Düsseldorf, Germany.

### Gene expression analysis

7.5 ml of EDTA whole blood were collected in BD vacutainer collection tubes. CD14^+^ blood monocytes were separated by AutoMACS Pro device (Miltenyi Biotec) using whole-blood CD14^+^ microbeads (Miltenyi Biotec). To preserve the RNA as well as possible, whole blood samples were processed immediately after collection. The CD14^+^ cell fraction was pelleted by centrifugation and directly processed for RNA isolation using the NucleoSpin RNA, Mini Kit for RNA Isolation (Machery & Nagel, Düren, Germany). All steps were performed on ice according to the manufacturer’s instructions. RNA samples were stored immediately at −70 °C until the use in RT qPCR experiments.

For the analysis of clock gene expression, primers for *PER1*,* PER2*,* PER3*, *CRY1*, *NR1D1*, *NR1D2* and *CRISPLD2* were designed according to following principles: 40–60% GC content, T_m_ of 50–60 °C, primer length ~ 20 bp, intron-spanning and resulting in an amplicon between 100 and 200 base pairs using Primer3 (all primer sequences in supplemental data S1). Primers were used in a final concentration of 100 to 300 nM each. Gene expression was analysed by Reverse Transcription Quantitative Polymerase Chain Reaction (RT-qPCR) using Takyon ROX SYBR 2X MasterMix dTTP blue and Takyon one step converter (Eurogentec). Amounts of RNA in each sample were adjusted by amplifying *β-Actin* as reference gene in the same run.

Data were corrected for efficiencies using the Pfaffl equation^28^ with early chronotypes determined as control group (Table 2). Each gene was analysed in triplicates. Replicates with mean Ct values with a standard deviation > 0.25 as well as replicated that did not reach an exponential phase and samples with less than two replicates were excluded from the analysis. For normalization *β-actin* was applied as a housekeeping gene. All RT-qPCR experiments were performed according to the MIQE-guidelines^29^.

### Statistical analysis

All statistical analyses were performed using SAS procedures (version 9.4; Cary, NC, USA). Participant characteristics are presented as mean (± SD) or median (Q1, Q3) values. Mann Whitney-U was used to detect statistical differences in gene expression of the selected clock genes because ΔCt values did not follow a normal data distribution. The test was used for the entire groups. P-values < 0.007, after Bonferroni correction for multiple testing, were considered a significant difference of expression of these genes in early and late chronotypes.

## Results

### Characteristics of participants

Participants included in this secondary analysis (*n* = 30) of the nutritional trial of the ChroNu study were on average 23 years old. The group of early chronotypes included 6 women and 5 men, while the group of late chronotypes consisted of 10 women and 9 men. Blood glucose and insulin values reflect metabolically healthy adults in both groups (Table 1).

The difference in MSFsc between individuals with early and late chronotypes was approximately 1:37 h: mm based on the MCTQ data (Table 1). Due to the timing of the morning blood sample the sleep duration was 6:19 h: mm (Q1 4:58; Q3 6:57) in persons with late chronotypes and 7:46 h: mm (Q1 7:04; Q3 8:20) in persons with early chronotypes in the night before the blood withdrawal. Individuals with late chronotypes had a later sleep onset whereas the wake-up times were comparable with individuals with early chronotypes (Table 1). Morning melatonin levels were not significantly different in individuals with late (median 32.9; Q1 28.3; Q3 44.1 ng/L) relative to individuals with an early chronotype (median 27.1; Q1 21.0; Q3 38.1 ng/L).

**Table 1:** Characteristics of participants by chronotype before the nutritional trial (*n* = 30) data are frequencies, mean (SD) or medians (Q1; Q3) depending on the data distribution.

General characteristics	Persons with early chronotype (*n* = 11)	Persons with late chronotype (*n* = 19)
Female/male, n	6/5	10/9
Age	23 (22; 24)	22 (21;23)
***Chronotype***		
MSFsc before intervention (h: mm) MSF actigraphy (h: mm) Sleep duration Sunday -> Monday (h) Sleep onset time Sunday (h: mm) Wake up time Monday (h: mm)	4:00 (3:32; 4:18) 3:42 (3:33; 4:12) 7:46 (7:04; 8:20) 22:30 (22:11; 22:28) 6:30 (5:50; 6:55)	5:37 (5:07; 6:07) 5:21 (5:01; 6:09) 6:19 (4:58; 6:57) 1:21 (0:29; 2:50) 6:45 (6:30; 6:52)
***Anthropometric and laboratory characteristics***		
BMI (kg/m^2^)	23.0 ± 2.5	22.9 ± 2.7
Skeletal muscle mass (kg)	25.3 ± 5.2	26.3 ± 6.8
Visceral adipose tissue (L)	0.4 (0.3; 0.9)	0.7 (0.4; 1.3)
Melatonin at 7 a.m. (ng/L) Insulin (µU/mL) Plasma glucose (mg/dL)	27.1 (21.0; 38.1) 6.7 ± 2.9 92.8 ± 4.3	32.9 (28.3; 44.1) 7.9 ± 3.7 91.8 ± 5.6

Abbreviations: n – sample size, BMI – body mass index, MSFsc – midpoint of sleep corrected at intervention, MSF actigraphy - midpoint of sleep on free days before intervention determined by actigraphy.

### Analyses of clock gene expression differences between persons with early and late chronotypes

**Fig. 2:**
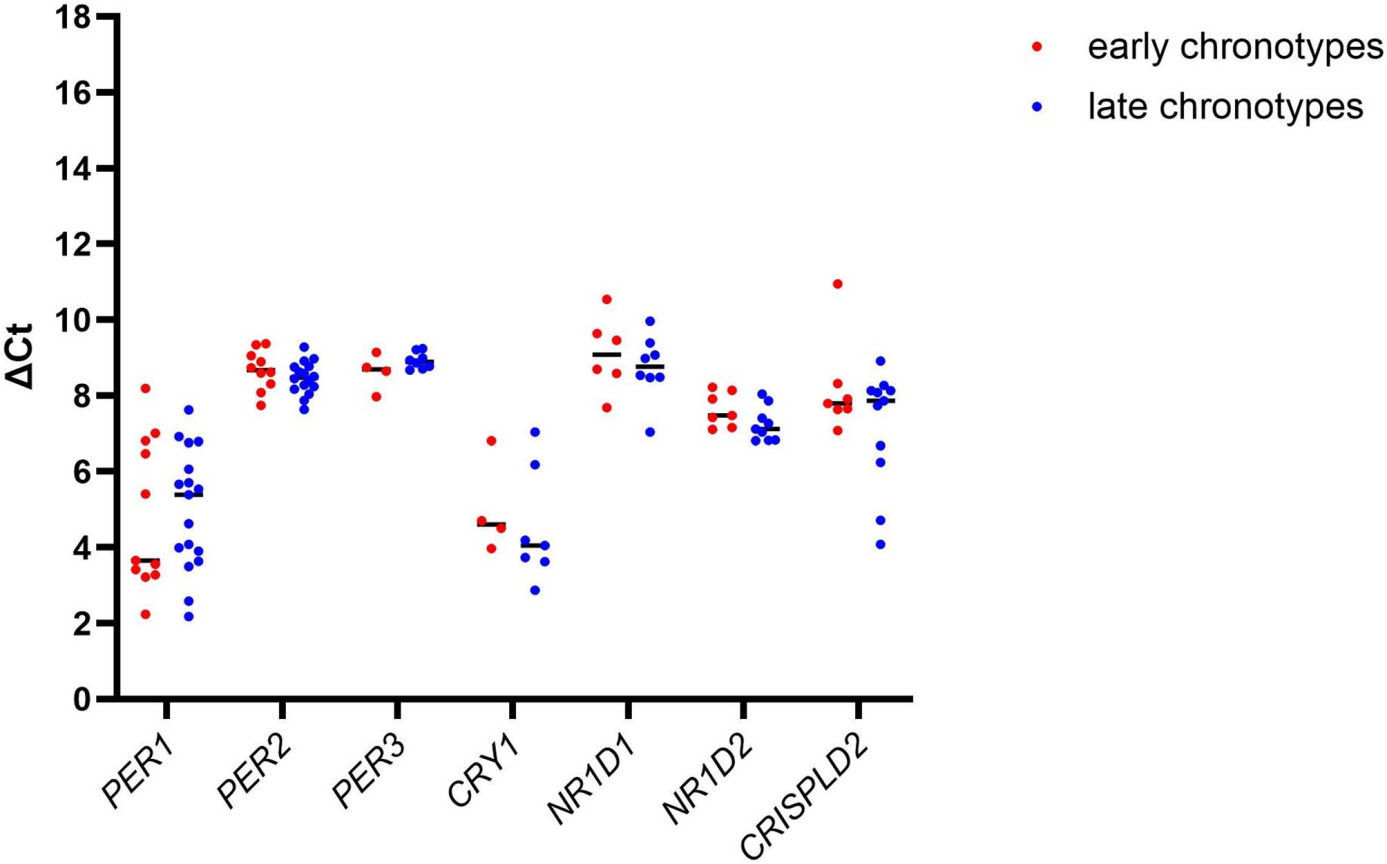
ΔCt for the seven analysed clock genes for persons with early and late chronotype.

The mean ΔCt values of individuals with early and late chronotypes show a high variability between participants of each group except for *PER2*,* PER3* and *NR1D2* (Fig. 2). To illustrate the intra-individual variation in the expression of the different clock genes, the mean ΔCt values were plotted for each single individual of early and late chronotype (Fig. S2). The high inter-individual variability shown for *PER1* and *CRY1*, and low inter-individual variability for *PER2* and *PER3* result in different intra-individual patterns of these clock genes. (see Figure S2). Since the inter-individual variability of some genes such as *PER1* and *CRY1* is seen across both groups of persons with early and late chronotypes, the overall clock gene profiles is relatively comparable between the chronotype groups (Fig. S2).

ΔCt values were calculated for each person of early (red dots) and late chronotypes (blue dots) normalized for the β-actin expression which was utilized as a reference gene in the same experiment. GraphPad Prism was used for illustration of the data. Abbreviations: nd – no difference.

The efficiency corrected ratio was about 1 for *PER1* and *PER2*. The ratio for *PER3*, *CRY1*,* NR1D1 and NR1D2* expression was slightly lower in individuals with late chronotypes than in individuals with early chronotypes whereas only *CRISPLD2* expression was slightly higher in persons with late than persons with early chronotypes (Table 2).

**Table 2:** Ratio of relative expression levels of clock genes in persons with early and late chronotypes. The efficiency corrected ratio was calculated according to the equation by Pfaffl, 2001 . For calculation the values of early chronotypes were used as the “control” condition. The PCR efficiency of each target gene and references gene were included in the calculation. Abbreviations: Ct - PCR cycle at which the fluorescence rises appreciably above the background fluorescence.

	mean Ct early chronotypes (*n* = 11)	mean Ct late chronotypes (*n* = 19)	mean PCR efficiency	Ratio_eff korr_
***PER1***	26.5	25.9	1.88	0.95
***PER2***	30.1	29.4	1.90	1.01
***PER3***	29.8	29.9	1.92	0.59
***CRY1***	26.5	26.2	1.91	0.78
***NR1D1***	30.8	30.4	1.84	0.78
***NR1D2***	28.5	28.4	2.00	0.68
***CRISPLD2***	29.3	27.9	1.89	1.55
***Beta-Actin***	21.6	20.9	1.97	1.00

Yet, no significant differences emerged between the two groups for any of the analysed genes (Table 3, all *p* ≥ 0.07).

**Table 3:** Differences in ΔCt values for mRNA levels of selected clock genes between persons with early and late chronotypes. Values are presented as mean ΔCt ± SD. Abbreviations: ΔCt – Ct value of respective target gene minus the value of the reference gene. Statistical analysis was performed by Mann-Whitney-U test.

	Mean ΔCt ± SD Per1	Mean ΔCt ± SD Per2	Mean ΔCt ± SD Per3	Mean ΔCt ± SD CRY1	Mean ΔCt ± SD NR1D1	Mean ΔCt ± SD NR1D2	Mean ΔCt ± SD CRISPLD2
**early chronotype** (*n* = 11)	4.84 ± 2.0	8.67 ± 0.6	8.62 ± 0.5	5.00 ± 1.3	9.10 ± 1.0	7.64 ± 0.5	8.20 ± 1.3
**late chronotype** (*n* = 19)	5.0 ± 1.6	8.47 ± 0.5	8.92 ± 0.2	4.53 ± 1.5	8.74 ± 0.9	7.25 ± 0.5	7.2 ± 1.6
**P-value**	0.67	0.28	0.21	0.40	0.40	0.07	0.65

## Discussion

To our knowledge, this is the first study to analyse clock gene expression at one single time point in persons with early and late chronotypes verified by actigraphy and a questionnaire. This study did not observe any difference in the expression levels of seven clock genes, *PER1*,* PER2*,* PER3*, *CRY1*, *NR1D1*,* NR1D2* and *CRISPLD2*, between individuals specifically selected for their early or late chronotype. Hence, our data did not support our hypothesis that due to circadian oscillation of clock genes, the peak of gene expression level might be phase-shifted in persons with late chronotypes in comparison to persons with early chronotypes.

The lack of phase-shifted clock gene expression in our study might be explained by the short half-life of expression^30^, yet RNA degradation was minimized by isolating monocytes immediately after blood collection, processing RNA on ice and analysing all seven clock genes expressed in CD14^+^ monocytes. Alternatively, the lack of different transcript levels might be explained by the circadian oscillation of clock genes. Current literature shows ambiguous findings regarding gene expression peaks throughout the day or in relation to the circadian rhythm which might be explained by the fact that they used other methodological approaches, e.g. by controlling conditions^13,31–33^, using other cell types^16,18,19,33^ or examining other sets of genes^14,17,19,32^. Wittenbrink et al. (2018) investigated a similar age group (22–30 years) in a small number of samples (*n* = 12). In contrast to our study, clock gene expression was analysed at regular time intervals so that a circadian oscillation was visible. From their data, the clock gene expression of genes that were used also in our study, *PER1*, *PER2*, *PER3*, *NR1D1*, *NR1D2* and *CRISPLD2* are increasing to/decreasing from the highest expression level at 7 a.m^13^. Overall, a comparison of these data with ours is hampered by the overall differences in the experimental settings such as RNA sample source (hair follicle cell, saliva, white blood cells), quantitative PCR settings and intervention conditions of subjects and lack of chronotype specific differences in gene expression levels.

In our study the inter-individual variability of some gene is larger than for the others. This might indicate that the amplitude of oscillations differs inter-individually or that discrepancies may be caused by environmental factors resulting in alternated oscillation of single genes^14,18^. We cannot rule out, that societal factors e.g. the misalignment caused by the early sample collection at 7 a.m. in persons with late chronotypes does alter gene expression. Additionally, clock genes might be sensitive to light exposure and therefore wake-up times. Novakova et al. showed that the peak of *PER1*, *PER2* and *NR1D1* expression correlates with midsleep point, but only *PER1* correlates significantly with the melatonin secretion^19^. Despite the difference in MSFsc, plasma melatonin levels at 7 a.m. did not differ between persons with early and late chronotypes. From dim light melatonin onset studies, it is known that plasma melatonin peak at 3:00 to 4:00 o’clock in the morning depending on the chronotype^39^ and decrease to below 2 pg/mL during daytime^40^. Therefore, the morning plasma melatonin level measured in this study might show that both groups of chronotypes are in a comparable circadian rhythm further supporting the lack of differences in clock gene expression. Furthermore, we cannot exclude cell-type specific expression patterns of clock genes. However, in this study a similar sample collection, was used as previously reported by Pivovarova et al. They also used CD14^+^ cell fraction of blood samples for analysis of clock gene expression^17^.

The absolute MSFsc might be crucial in these types of analyses. According to reports of Roenneberg et al. (2007) young adults around 20 years of age have the latest chronotypes^34^. Nonetheless, in our study group only very few persons with extreme early or late chronotypes were included, presumably reflecting the difficulties to recruit these individuals into a study requiring early blood sample and consumption of meals at assigned times of the day, as done in this nutrition trial^22^. Most participants included in this secondary analysis showed moderate early and moderate late MSFsc. Nonetheless, this study managed to include two samples of diverging chronotype as reflected in a remarkable difference in MSFsc. Of note, most previous studies have applied a median split to distinguish persons with early and late chronotypes^9,11,16,35–37^ or did not separate participants according to their chronotype^13–15,17^ to study clock gene expression. One study used the MCTQ to classify participants in five groups of chronotypes (extreme early, slight early, normal or moderate, slight late, and extreme late type). However, this study focussed on the detection of clock gene variants rather than on quantification of mRNA levels^38^. Our observation that clock gene expression levels did not differ at 7.am. is supported by a recent publication reporting no difference in the expression level between healthy persons of both sexes with early (*n* = 8) and late chronotypes (*n* = 6) for the two core clock genes, *PER1* and *CRY1.* However, in that study again chronotype groups were defined by median-split in morning and evening types. Of note, expression of these genes did also not differ at 7 p.m^37^.

It should be noted that assignment of gene expression to a chronotype may be hampered by clock gene variants which might also quantitatively differ in expression level. We cannot exclude that the similar clock gene expression level is altered by clock gene variant expression or interactions of clock gene variants. So far, gene variants were reported qualitatively in large cohorts. For example, *PER3*^39–42^ were associated with sleep alterations and metabolic diseases such as type 2 diabetes mellitus. RevErbα and β (*NR1D1* and *NR1D2*) were associated with glucose homeostasis^36,43,44^. *CRY1* gene variants were previously associated with carbohydrate intake and insulin resistance^45^. The authors even recommend persons with gene variants in *CRY1*, *CLOCK* or the gene for the melatonin receptor *MNTR1b* to focus on diet and normal sleep duration^46^.

### Strengths and limitations

The strength of our study is that we were able to analyse accurately estimated early and late chronotypes and were able to show the difference in sleep onset on the day before blood sample collection by actigraphy in our participants. Moreover, young adults of about 20 years do have the largest shift in chronotype^47^. Therefore, a difference should be most likely be seen in this age group.

Limitations to our study are that this secondary analysis presents a gene expression analysis of a very small number of participants. In contrast to Wittenbrink et al. (2018), Akashi et al. (2010) and Takahashi et al. (2018), who analysed clock gene expression quantitatively only in young healthy men, we analysed samples from both biological sexes. This might be both strength and limitation of our study as this shows a broader picture of chronotype differences in gene expression in both men and women but also reduced the samples in each biological sex group.

Another limitation might be that we analysed RNA from one single time point per person only, because clock gene expression might oscillate differently in persons with early and late chronotypes. The gene expression analysis was not originally within the scope of the ChroNu study, but precisely because we only analysed a single time point, we were able to show that such a snapshot is not sufficient for the analysis of different clock gene expression in persons with early and late chronotypes. Moreover, RNA was isolated from the CD14^+^ fraction only. To rule out a cell-specific effect, additional RNA samples from other cell types, e.g. hair follicle cells might be analyzed in future studies.

Future studies on larger samples with both sexes should recruit individuals with more extreme chronotypes. Repeated melatonin measurements parallel to RNA sample collection might further support the clock gene expression levels.

## Conclusion

In this secondary analysis of young healthy adults of both sexes, we observed no chronotype-specific difference in gene expression level of *PER1*, *PER2*, *PER3*, *CRY1*, *NR1D1*, *NR1D2* and *CRISPLD2* in an early morning blood sample withdrawn at 7 a.m. Differences in clock gene expression – i.e. the hypothesis of this paper - might only be visible when altered circadian oscillations of clock gene expression are monitored in several time intervals or larger sample sizes.

## Electronic supplementary material

Below is the link to the electronic supplementary material.

Supplementary Material 1

## Data Availability

The datasets used and/or analysed during the current study available from the corresponding author on reasonable request.
