# Supplementary material for: Morning clock gene expression in young adults of early and late chronotypes
Source: Sci Rep. 2025 Jul 23;15:26709. doi: 10.1038/s41598-025-12423-7 (PMC12283915; doi:10.1038/s41598-025-12423-7)
Supplement: Supplementary file 1 — Supplementary Material 1 [file 41598_2025_12423_MOESM1_ESM.docx]

**Supplement S1: Primer Sequences for RTqPCR**

| **gene** | **forward primer 5‘🡪 3‘** | **reverse primer 5‘🡪 3‘** | **length (bp)** |
| --- | --- | --- | --- |
| *PER1* | aggtacctggagagctgcaa | ttcttggtccccacagagac | 131 |
| *PER2* | ctggccatccacaaaaagat | cctcccaatgatgaaggaga | 159 |
| *PER3* | tcctggcgtcttctcacttt | tcataccgtgcagctctttg | 124 |
| *NR1D1* | ctgggaggatttctccatga | ttcacgttgaacaacgaagc | 168 |
| *NR1D2* | cagcaatgtcgcttcaaaaa | tggtcttcattgcactttgc | 124 |
| *CRY1* | caggttgtagcagcagtgga | gactaggacgtttcccacca | 141 |
| *CRISPLD2* | tcactgaagcaacagcatcc | aggaacattttgggcaactg | 146 |
| *β-actin* | gatgagattggcatggcttt | caccttcaccgttccagttt | 100 |

**Supplement S2: clock gene expression level in each subject**


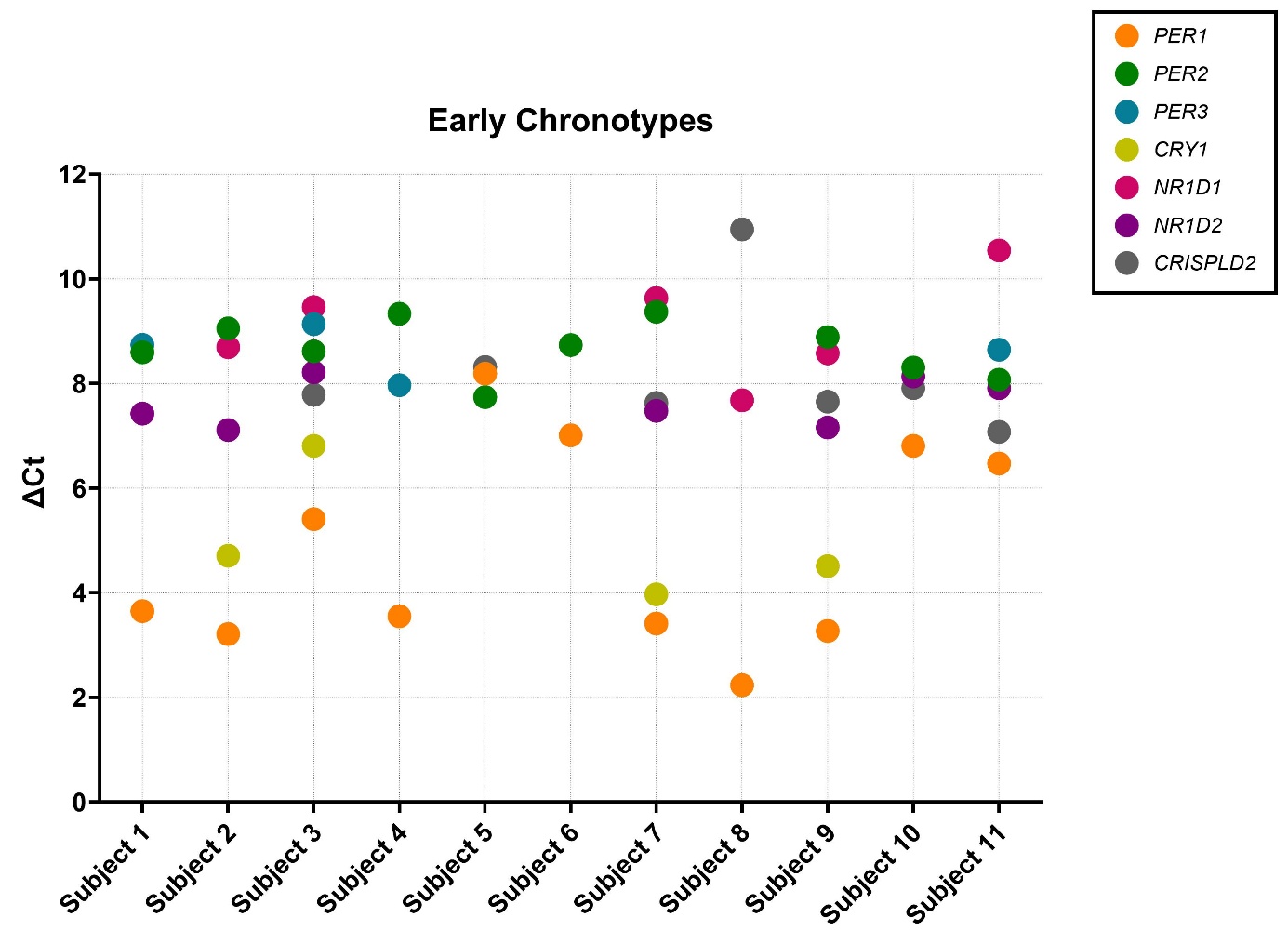


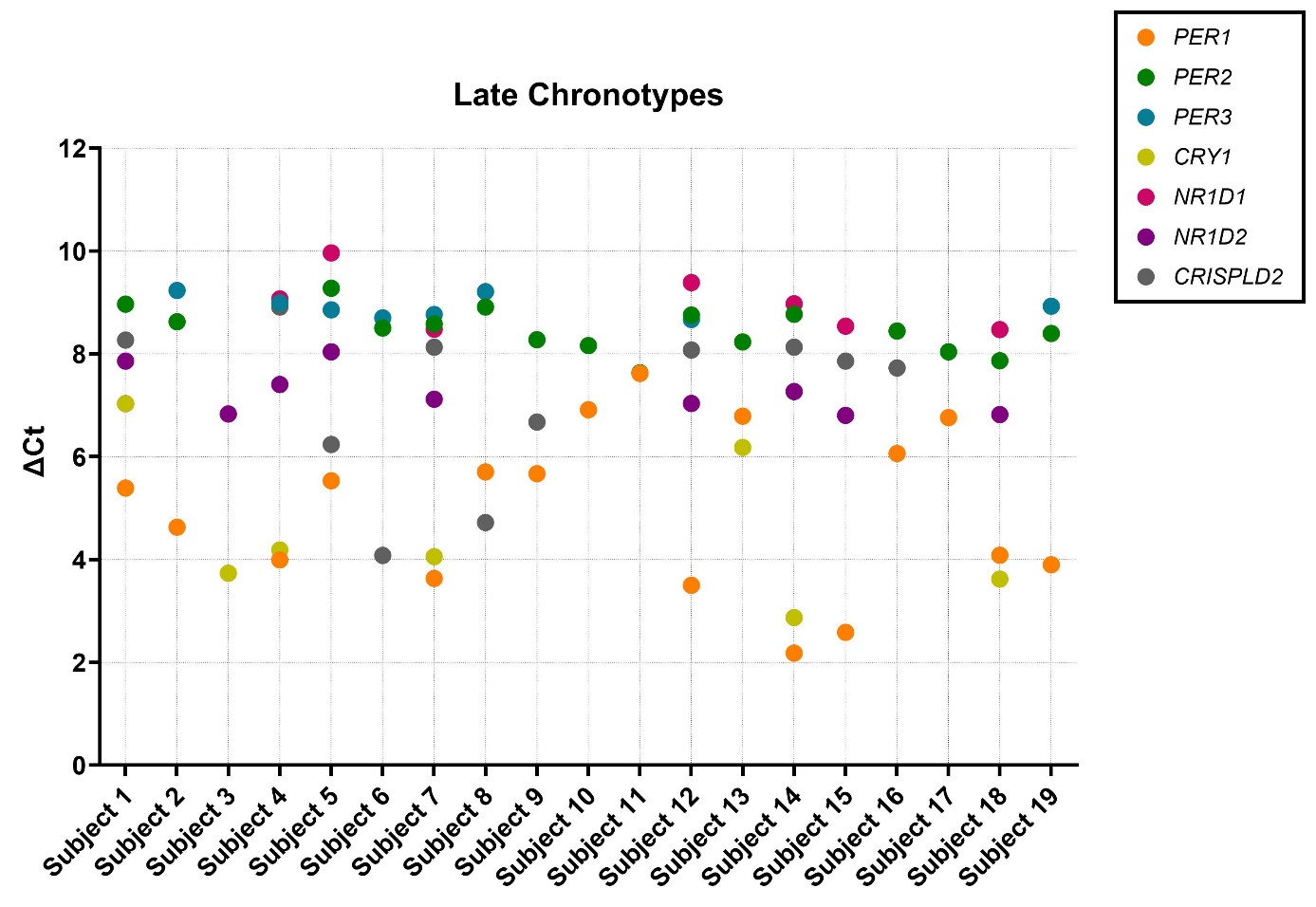


**Fig. S2**: ΔCt for the seven analysed clock genes for each person with early or late chronotype. ΔCt values were calculated for each person of early (upper graph) and late chronotypes (lower graph) normalized for the *β-actin* expression which was utilized as a reference gene in the same experiment. For some subjects, experiments were not included for all clock genes because the variability of replicates was too high. ΔCt values for *PER1* and *CRY1* showed the highest variability whereas *PER2* and *PER3* are more constant (for further details in the discussion).

**Supplement S3:** **Schematic overview of expected clock gene expression level at 7 a.m.**


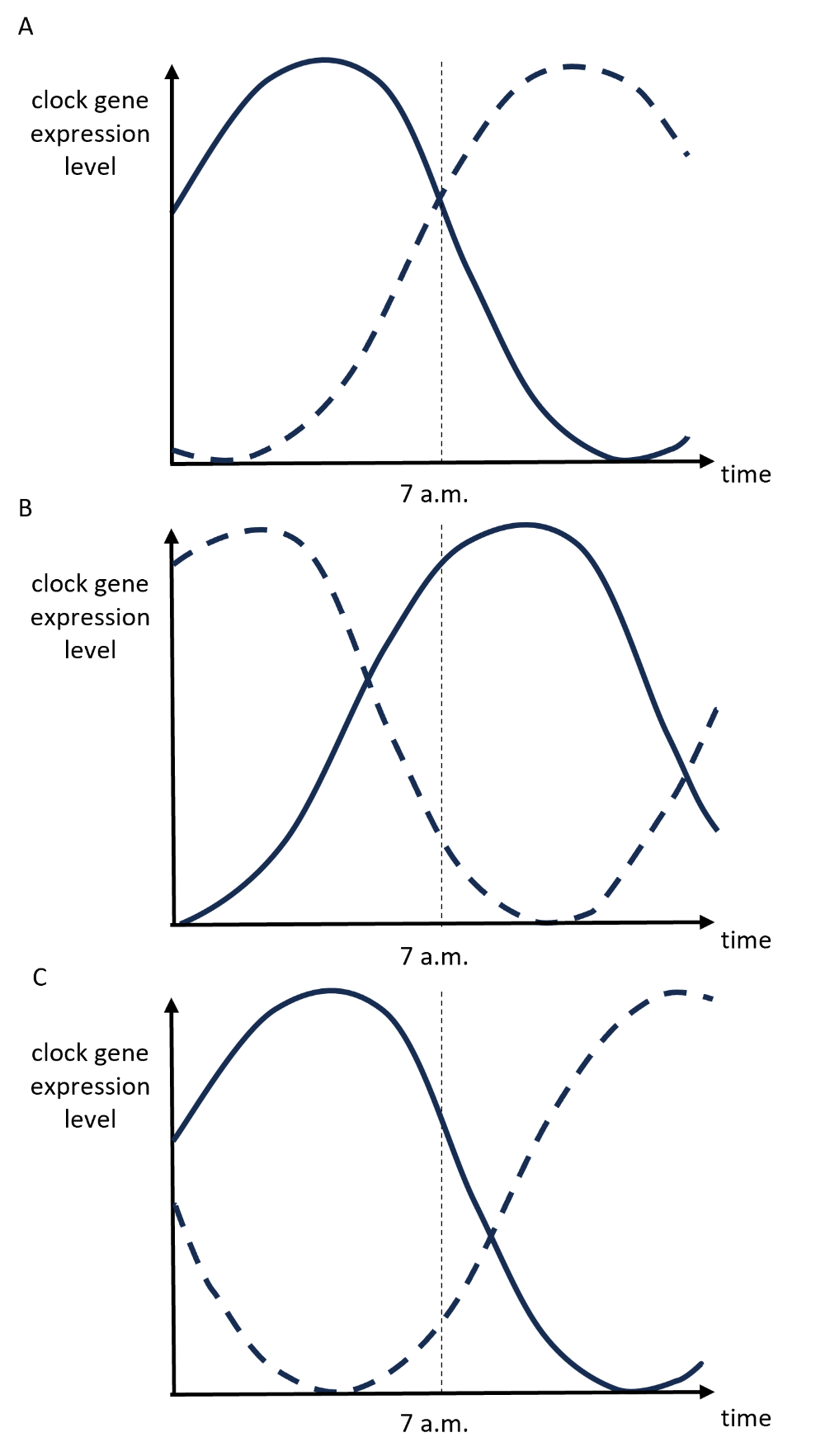


Fig. S3: The literature contains varying information on the expression levels of the clock genes. Because of the oscillating expression levels, a similar or a phase-shifted expression level can be expected in persons with early and late chronotypes at 7 a.m. Only references that specified actual times of day or melatonin values were considered here (see discussion for details). A: Data from some references suggest that the expression levels of *PER1-3*, *CRY1* and *NR1D1* are likely similar at 7 a.m. in persons with early and late chronotype probably because in persons with late chronotypes, the peak of gene expression has not yet been reached, whereas in persons with early chronotypes it is already falling again. B and C: Other references indicate different peak expressions of *PER1-3*, *CRY1*, *NR1D1*, *NR1D2* and *CRISPLD2*, suggesting a phase-shifted expression in persons with early (solid line) or late chronotype (dashed line) in one direction or the other.
